# Metabolic Overlap between Alzheimer’s Disease and Metabolic Syndrome Identifies the *PVRL2* Gene as a New Modulator of Diabetic Dyslipidemia

**DOI:** 10.3390/ijms24087415

**Published:** 2023-04-18

**Authors:** Montse Guardiola, Gerard Muntané, Iris Martínez, Lourdes Martorell, Josefa Girona, Daiana Ibarretxe, Núria Plana, María J. Bullido, Elisabet Vilella, Josep Ribalta

**Affiliations:** 1 Unitat de Recerca en Lípids i Arteriosclerosi, Departament de Medicina i Cirurgia, Universitat Rovira i Virgili, 43201 Reus, Spain; 2 Institut d’Investigació Sanitària Pere Virgili-CERCA, 43204 Reus, Spain; 3 Centro de Investigación Biomédica en Red de Diabetes y Enfermedades Metabólicas Asociadas, CIBERDEM-Instituto de Salud Carlos III, 28029 Madrid, Spain; 4 Hospital Universitari Institut Pere Mata, 43206 Reus, Spain; 5 Genètica i Ambient en Psiquiatria, Departament de Medicina i Cirurgia, Universitat Rovira i Virgili, 43201 Reus, Spain; 6 Centro de Investigación Biomédica en Red en Salud Mental, CIBERSAM-Instituto de Salud Carlos III, 28029 Madrid, Spain; 7 Unitat de Medicina Vascular i Metabolisme, Servei de Medicina Interna, Hospital Universitari Sant Joan de Reus, 43204 Reus, Spain; 8 Centro de Biología Molecular “Severo Ochoa” (C.S.I.C.-U.A.M.), Universidad Autónoma de Madrid, 28049 Madrid, Spain; 9 CIBERNED, Center for Networked Biomedical Research on Neurodegenerative Diseases, Carlos III Institute of Health, 28029 Madrid, Spain; 10 Instituto de Investigación Sanitaria del Hospital Universitario La Paz, IdiPAZ (Hospital Universitario La Paz—Universidad Autónoma de Madrid), 28029 Madrid, Spain

**Keywords:** Alzheimer’s disease, diabetes, lipid, *PVRL2*

## Abstract

Background: Alzheimer’s disease (AD) and type 2 diabetes mellitus (T2DM) share metabolic alterations such as abnormal insulin and lipid metabolism and have some common genetic factors such as *APOE* genotype. Taking this into account, we hypothesized that we could identify common genetic factors involved in the development of diabetes and cardiovascular diseases. Methodology: We first genotyped 48 single nucleotide polymorphisms (SNPs) previously associated with AD in a cohort composed of 330 patients with cognitive impairment (CI) to assess their association with plasma lipids. Second, we conducted pleiotropy-informed conjunctional false discovery rate (FDR) analysis designed to identify shared variants between AD and plasma lipid levels. Finally, we used the SNPs to be found associated with lipid parameters and AD to search for associations with lipoprotein parameters in 281 patients with cardiometabolic risk. Results: Five SNPs were significantly associated with lower levels of cholesterol transported in remnant lipoprotein particles (RLPc) in subjects with CI; among these SNPs was the rs73572039 variant in *PVRL2*. Stratified QQ-plots were conducted on GWAS designed for AD and triglycerides (TG). The cross-trait analysis resulted in a total of 22 independent genomic loci associated with both AD and TG levels with a conjFDR < 0.05. Among these loci, two pleiotropic variants were located in *PVRL2* (rs12978931 and rs11667640). The three SNPs in *PVRL2* were significantly associated with RLPc, TG, and number of circulating VLDL and HDL particles in subjects with cardiometabolic risk. Conclusions: We have identified three variants in *PVRL2* that predispose individuals to AD that also influence the lipid profile that confers cardiovascular risk in T2DM subjects. *PVRL2* is a potential new modulating factor of atherogenic dyslipidemia.

## 1. Introduction

Alzheimer’s disease (AD) and diabetes mellitus (DM) are among the most common diseases associated with aging [1,2]. These diseases share common elements and may be viewed as fundamentally similar disorders that differ in the magnitude of specific traits, primarily the affected tissues and the age of onset [3].

AD is the most common cause of dementia. However, it has also been described as a metabolic disease since it causes losses in the ability of the brain to efficiently utilize glucose for energy production [4]. The consequences of insulin resistance in the brain are similar to those in other organs and tissues.

In 2005, Suzanne de la Monte introduced the term “type 3 diabetes mellitus” (T3DM) in reference to AD [5]. The term T3DM accurately reflects the fact that AD represents a form of diabetes that selectively involves the brain and has molecular and biochemical features that overlap with both type 1 and type 2 diabetes mellitus (T1DM and T2DM, respectively). Insulin levels and the expression of insulin receptors are decreased in the brains of AD patients [6,7], suggesting that there is progressive brain insulin resistance. It has also been proposed that aging is associated with a dysfunctional energy metabolism that, in the brain, may manifest as T2DM [8].

AD and T2DM also share a common genetic background; the clearest example of this is apolipoprotein E (apoE). The E4 isoform of APOE is the most established genetic risk factor for AD [9]. ApoE plays a central role in lipid metabolism and is recognized by the hepatic receptors and heparin sulfate proteoglycans involved in the clearance of very-low-density lipoproteins (VLDLs), chylomicrons and their remnants [10]. Remnant lipoprotein particles (RLPs) are derived from and accumulate in circulation due to impaired lipid metabolism triggered by insulin resistance. Cholesterol transported in RLPs (RLPc) increases the risk of atherosclerosis [11,12,13]. The circulating levels of RLPc are elevated in T2DM patients [14], although this feature has not yet been studied in AD patients.

In addition, pleiotropy studies designed to detect cross-phenotype genetic associations between cardiovascular risk factors and AD have demonstrated that the genetic enrichment in AD is limited to those single-nucleotide polymorphisms (SNPs) significantly associated with lipid parameters. These findings highlight the involvement of the locus on chromosome 19 that contains the *TOMM40/APOE/APOC1/PVRL2* genes [15,16].

Since AD and T2DM share metabolic alterations such as abnormal insulin lipid metabolism and have some common genetic background, our hypothesis is that we could identify common genetic factors involved in the development of AD and the diabetic dyslipidemia found in diabetic subjects. To accomplish this, we employed two different approaches. First, in a cohort composed of patients with cognitive impairment (AD and mild cognitive impairment (MCI) patients), we genotyped 48 SNPs previously associated with AD and searched for potential associations with circulating lipids. Second, we conducted pleiotropy-informed conjunctional false discovery rate (FDR) analysis using previously published GWAS designed to identify shared variants between AD and lipid levels. Finally, we assessed the associations between the SNPs found in *PVRL2* located in *TOMM40*/*APOE*/*APOC1* loci to be associated with both traits and lipids and cardiovascular risk factors in a population with high cardiovascular risk.

## 2. Results

To identify new candidate genes that associate with the characteristic lipids of atherogenic dyslipidemia that we find in patients with diabetes, we followed two strategies:

- genotype 48 SNPs in AD candidate genes previously studied in a cohort with cognitive impairment, and look for associations with lipids.
- carry out a pleiotropy study between AD and TG.

### 2.1. Association between SNPs and Lipids in the Cognitive Impairment Population

First, we studied a group of 330 subjects with cognitive impairment. Among these individuals, 146 were diagnosed with MCI and 184 were diagnosed with AD. Baseline characteristics are depicted in Table 1. There were no statistically significant differences in lipid parameters between the groups; thus, we decided to study all the subjects together.

Forty-eight SNPs previously associated with AD (Supplemental Table S1) were genotyped, and all of them followed Hardy–Weinberg equilibrium. Among these SNPs, five were significantly associated with lower levels of RLPc (Table 2). Carriers of the rare allele in rs1789072 (in *DSC1*) had an 11% decrease in RLPc levels (*p* = 0.047). Carriers of the rare allele in rs3025786 (in *PSEN1*) had a 16% decrease in RLPc levels (*p* = 0.012). Carriers of the rare allele in rs73572039 (in *PVRL2*) had a 14% decrease in RLPc levels (*p* = 0.009) compared to those who did not carry the rare allele. Individuals who were homozygous for the rare allele of rs3788428 (in *PLA2G3*) presented 46% (*p* = 0.015) lower RLPc levels, and those homozygous for the rare allele of rs1049296 (in *TF*) had a reduction of 16.5% (*p* = 0.05) compared to common allele carriers.

### 2.2. Shared Genetic Architecture between AD and TG Levels

**Figure 1:**
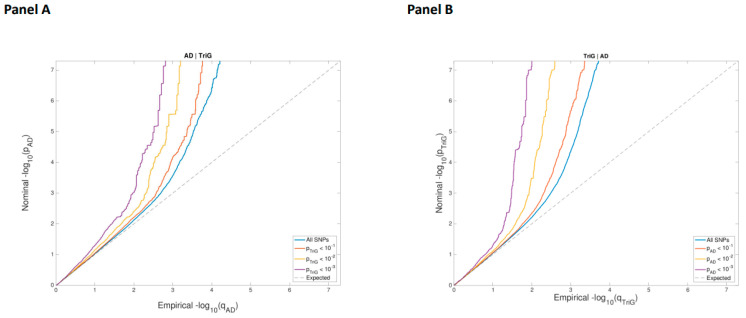
Stratified QQ-plots of AD conditioned on increasing triglyceride levels (**A**) and TG conditioned on AD (**B**).

Second, we used genome-wide association studies to identify the genetic variants jointly associated with AD and TG [17,18]. First, stratified QQ-plots showed a strong leftward deflection from the null expectation when conditioning AD on increasing levels of association with TG levels, which is consistent with a shared genetic architecture between both traits (Figure 1). The cross-trait analysis resulted in a total of 22 independent genomic loci associated with both AD and TG levels with a conjFDR < 0.05 (Supplemental Table S2). Among these loci, two pleiotropic variants were located in *PVRL2* (rs12978931 and rs11667640).

*PVRL2* was identified as a locus associated with lipid parameters through the two previous approaches. Thus, our next step was to confirm this association in a cohort of patients with high cardiovascular risk and to assess the modulatory effect of *PVRL2* variants on lipoprotein metabolism.

### 2.3. Association between PVRL2 SNP Variants and Lipoprotein Parameters in a Population with Cardiometabolic Risk

The cohort with cardiometabolic risk was composed of 281 middle-aged subjects with dyslipidemia; of these subjects, 67% presented with type 2 diabetes, 52% presented with obesity, and 75% presented with metabolic syndrome (Table 3). We genotyped the three SNPs previously identified in *PVRL2* (rs73572039, rs12978931 and rs11667640). The minor allele frequencies were similar to those described in European ancestries, and all the genotype distributions followed Hardy–Weinberg equilibrium.

The associations between the three *PVRL2* variants and the complete profile of six lipoprotein subclasses assessed by NMR are shown in Table 4. Carriers of the rare allele of rs73572039 presented lower circulating TG levels (−17%) and lower RLPc levels (−10%) than those who were not carriers (*p* = 0.032 and *p* = 0.049, respectively). These associations were accompanied by decreased levels of the lipoproteins that transport most of the TG in circulation, the VLDL particles. Carriers of the rs73572039 rare allele presented lower total VLDL particle numbers (−25%, *p* = 0.007), and this was also found in all their subclasses, namely, the large VLDL (−27%, *p* = 0.009), medium VLDL (−24%, *p* = 0.007), and small VLDL particles (−28%, *p* = 0.007). This lipoprotein profile was also accompanied by a 6% decrease in small HDL particles (*p* = 0.016).

When analyzing results for rs12978931, we found that those subjects who were homozygous for the rare allele presented with an increase of 146% in circulating TG levels and 73% higher RLPc levels compared to carriers of the common allele (*p* = 0.001 and *p* = 0.045, respectively). These associations were accompanied by an increased number of VLDL particles. Total VLDL particles were 111% higher in subjects homozygous for the rare allele of rs12978931 (*p* = 0.003). These findings are similar to what was found for all the VLDL subclasses: a 97% increase in large VLDL (*p* = 0.003), 106% increase in medium VLDL (*p* = 0.003), and 113% increase in small VLDL particles (*p* = 0.003). This lipoprotein profile was accompanied by a 17% increase in small HDL particle number (*p* = 0.039).

Finally, subjects who were homozygous for the rare allele in rs11667640 presented with a 57% reduction in HDL cholesterol levels (*p* = 0.006) and a 180% increase in RLPc levels (*p* = 0.030) compared with wild-type individuals.

## 3. Methods

### 3.1. Population with Cognitive Impairment

We studied a sample of 330 participants with cognitive impairment recruited between 1998 and 2002 at the Unitat de Memòria-Alzheimer at the Hospital Universitari Institut Pere Mata in Reus, Spain. This cohort has been previously described elsewhere [19].

The participants were adults of 55 to 95 years of age who were diagnosed with mild cognitive impairment (MCI, n = 146) or Alzheimer-type dementia (AD, n = 184). The diagnostic criteria used were those included in the DSM-IV and the ICD-10, in addition to the NINCDS-ADRDA for AD and the Petersen criteria for MCI. Participants presenting with vascular (using the Hachinski scale), frontotemporal (following the Lund-Manchester criteria) and Lewy body (following the McKeith criteria) dementia cases were excluded as previously described [19].

The study was approved by both the Committee of Research at the Hospital Universitari Institut Pere Mata and the Ethical Committee at the Hospital Universitari Sant Joan de Reus on 9 April 1997. All participants gave their written consent to participate. Data were coded for anonymity in accordance with current Spanish law on biomedical research.

### 3.2. Population with Cardiometabolic Risk

We included 281 patients at high risk of cardiovascular diseases because of hyperlipidemia, obesity, metabolic syndrome or diabetes among those subjects attending the Vascular Medicine and Metabolism Unit of the Hospital Universitari Sant Joan de Reus. The Adult Treatment Panel III criteria were used to define the presence of metabolic syndrome and T2DM. Patients on lipid-lowering drugs had a washout period of 6 weeks (8 weeks if they were receiving fibrates). Anamnesis and physical examination were recorded.

The Ethical Committee at the Hospital Universitari Sant Joan de Reus approved this study (code 200/2018), and all patients gave their written consent to participate. Data were coded for anonymity in accordance with current Spanish law on biomedical research.

### 3.3. Blood Sample Collection and Storage

A blood sample was obtained from each individual after overnight fasting. Upon laboratory arrival (less than 2 h from extraction), samples were immediately centrifuged to separate plasma from cells. Plasma was stored at −80 °C until biochemical analyses were performed. Genomic DNA was extracted from peripheral blood mononuclear cells using the Gentra Puregene BloodKit (QIAGEN Iberia S.L., L’Hospitalet de Llobregat, Barcelona, Spain) according to the manufacturer’s instructions. The extraction was carried out at the Biobank of the Institut d’Investigació Sanitària Pere Virgili (IISPV; Reus, Tarragona, Spain).

### 3.4. Biochemical Analyses

In the population with cognitive impairment, total cholesterol (TC) levels were measured following standard enzymatic biochemical techniques. In the high cardiovascular risk population, total cholesterol, HDL cholesterol (HDLc), LDL cholesterol (LDLc), triglycerides (TG), apolipoprotein B100 (APOB100) and apolipoprotein AI (APOAI) levels were measured using standard enzymatic and colorimetric techniques adapted to a Cobas Mira autoanalyzer (Roche Diagnostics, Spain). The cholesterol contained in the remnant-like particles (RLPc) was measured in both populations from plasma samples using RLP cholesterol assay kits (Jimro-II, Japan Immunoresearch Laboratories, Tokyo, Japan) according to the manufacturer’s instructions [20].

### 3.5. Lipoprotein Profile Characterization

Samples from the high cardiovascular risk population were subjected to the Liposcale test. This advanced lipoprotein test provides the distribution of 9 lipoprotein plasma subclasses; this method allows for a more detailed and informative lipid profile that is based on 2D diffusion-ordered 1-H^+^ nuclear magnetic resonance (NMR) spectroscopy. This method adds diffusion coefficients to classic NMR determinations to provide a direct measure of mean particle size and number for each lipoprotein fraction. This technique also provides the mean particle size and concentration of three subfractions of each lipoprotein class (large, medium and small) [21].

### 3.6. Genetic Strategy and Genotyping

**Figure 2:**
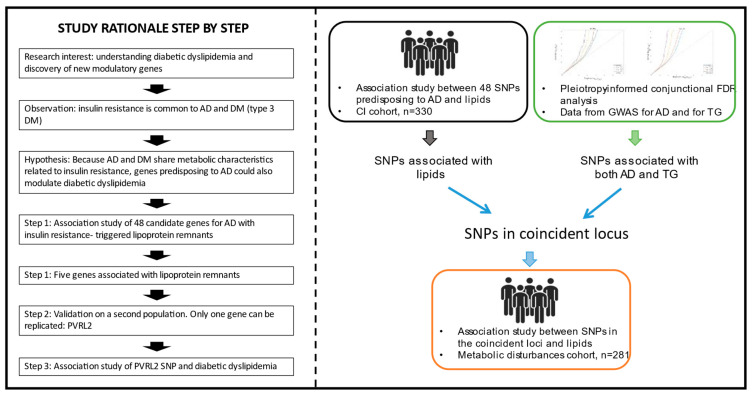
Schematic of the strategy followed to identify new genes associated with AD and metabolic disorders.

Figure 2 shows a schematic of the strategy we followed to identify new genes associated with AD and metabolic disorders. We carried out two different strategies.

First, we genotyped a total of 48 SNPs from 33 genes that predispose patients to AD in the cognitive impairment population. These SNPs were selected based on the literature [22,23,24,25,26,27,28,29,30,31,32,33,34] and are summarized in Supplemental Table S1. All genotypes were determined using TaqMan genotyping in an AbiPrism 7900HT Sequence Detection System using TaqMan SNP Genotyping assays (Applied Biosystems, Madrid, Spain) and TaqMan Genotyping Master Mix (Applied Biosystems, Madrid, Spain). Data acquisition and allelic discrimination analysis were performed using SDS v2.4 software (Applied Biosystem, Madrid, Spain).

Second, we performed a conjunctional FDR analysis. GWAS summary statistics on AD were obtained from a previous study [17] that comprised association analyses of a total of 71,880 patients and 383,378 control individuals of European ancestry. The summary statistics on TG levels were obtained from a previous study [18] that included 188,577 individuals of European descent. GWAS summary statistics were referenced to a set of 9,546,816 SNPs generated from the 1000 Genomes Project (1KGP, https://www.internationalgenome.org/). SNPs that were nonbiallelic, without rsIDs, duplicated, or with strand-ambiguous alleles were removed. SNPs with INFO scores < 0.9 in the summary statistics files, those mapping to the extended major histocompatibility complex (MHC, genomic position in hg 19; chr6: 25,119,106–33,854,733) and the 8p23.1 region (chr8: 7,200,000–12,500,000), which are prone to rearrangements [35], SNPs located on chromosomes X and Y and mitochondrial DNA, and SNPs with sample sizes 5 standard deviations away from the mean were also filtered out. Finally, a common set of 3,203,544 SNPs was kept in both datasets. All ORs and betas from the summary statistics were transformed to z scores. Then, we used pleioFDR (https://github.com/precimed/pleiofdr) to identify genetic loci jointly associated with two phenotypes, setting a conjFDR level of 0.05 for each phenotypic pairwise comparison. We evaluated the directional effects of the loci shared between AD and TG levels by comparing their z scores. All *p* values were adjusted for standard genomic control (GC). The European populations from the 1KGP were used as the reference panel for the computation of the linkage disequilibrium (LD) structure between SNPs. To define distinct genomic loci, we merged any physically overlapping lead SNPs (LD blocks < 250 kb apart), and the borders were defined by identifying all SNPs in LD (r2 ≧ 0.1) with one of the independent significant SNPs in the locus. The region containing all these candidate SNPs was defined as a single independent genomic locus, and the most significant SNP within the region was selected as the lead SNP.

Finally, following the two previous genetic strategies, we identified three SNPs in PVRL2 (rs73572039, rs12978931, and rs11667640). These three SNPs were genotyped in the high cardiovascular risk population using TaqMan genotyping as previously described to evaluate if these SNPs are also associated with cardiometabolic factors in a population at risk.

### 3.7. Statistical Analysis

Allele frequencies and Hardy–Weinberg equilibrium of SNP distributions were estimated by chi-squared analyses.

Associations between lipid variables and genotypes were evaluated using either Mann–Whitney or ANOVA tests depending on the distribution of the variables. Age, sex, APOE genotype and body mass index (BMI) were considered covariates.

SPSS package version 23.0 (IBM, Madrid, Spain) was used throughout the study. A value of *p* < 0.05 was considered significant.

## 4. Discussion

Complex diseases, such as AD and T2DM, are caused by the interplay of genetic, environmental and lifestyle factors, most of which have yet to be identified. Accumulating evidence suggests that different complex diseases are genetically correlated, sharing genetic risk bases [36]. Thus, identifying these common genetic factors can help us discover new genes involved in the development of complex diseases. Considering that AD and T2DM share a common metabolic background, we hypothesized that we could identify novel genes modulating the characteristic lipid profile of T2DM using different genetic approaches. First, we explored the association between SNPs previously linked to AD and their effect on lipid parameters in a population of subjects with cognitive impairment. Second, we performed a pleiotropy-based analysis designed to identify shared genetic variants between AD and TG levels. Both strategies indicated *PVRL2* as a link between AD and lipid parameters characteristic of atherogenic dyslipidemia present in subjects with T2DM. Finally, we genotyped the identified variants in a population with high cardiometabolic risk to confirm and deepen its modulatory capacity on lipids and lipoproteins.

AD is the most common neurodegenerative disease, and the prevalence of developing MCI or AD is higher in individuals with T2DM than in individuals without diabetes [37]. However, the common mechanisms that underlie T2DM and cognitive impairment remain unclear. T2DM is mainly characterized by insulin resistance, which triggers alterations in lipid metabolism, also known as atherogenic dyslipidemia, that confer an elevated risk of cardiovascular conditions [38]. Atherogenic dyslipidemia is characterized by a combination of high TG levels and low HDLc levels, accompanied by nonpathological or moderately elevated LDLc levels [39]. Advanced NMR analysis of the lipoprotein profile reveals that atherogenic dyslipidemia is characterized by increased concentrations of large VLDL particles and smaller LDL and HDL particles [21]. In addition, the number of remnant IDL particles and concentrations of cholesterol transported in RLPs are increased by atherogenic dyslipidemia and are independent cardiovascular disease risk factors [40].

The relationship between lipids and AD has been previously and widely explored. The role of cholesterol in brain functions is clear, and several AD-risk SNP-related genes cluster in cholesterol and lipid pathways [41]. Subjects suffering from AD and subjects with T2DM share an abnormal lipid profile featuring increased plasma TG levels, lower HDLc levels [42] and a predominance of proatherogenic small dense LDL particles [43,44,45]. In addition, the E4 allele of the *APOE* gene is the most established genetic risk factor for AD [9]. Apolipoprotein E plays a central role in lipid metabolism since it is recognized by the hepatic receptors and heparin sulfate proteoglycans involved in the clearance of VLDL, chylomicrons and their remnants [10].

Several genetic strategies have been carried out in recent years to facilitate the identification of molecular players in AD. GWASs [17,46], targeted polygenic studies [47,48], multiomic strategies [49], and even genetic pleiotropy approaches have been performed [16], and a strong relationship between circulating lipids and genetic factors related to AD has been described [47]. However, other studies have found no conclusive relationship between lipids and AD using Mendelian randomization [50]; therefore, this is a topic that still requires attention.

Two recent pleiotropic approaches evaluating the effects of genetic variants associated with cardiovascular and metabolic risk factors on cognitive impairment conditions have identified that circulating lipid parameters in subjects with cognitive impairment are associated with the *APOE*-*TOMM40*-*APOC1*-*PVRL2* loci on chromosome 19 [15,16].

Importantly, in the present study, we have described that rs73572039, rs12978931 and rs11667640 in *PVRL2* are associated with those lipid parameters that define atherogenic dyslipidemia, such as the number of VLDL particles and TG, HDLc and RLPc levels both in subjects with mild cognitive impairment and in subjects with high cardiometabolic risk. These findings suggest that *PVRL2* should be considered when assessing cardiovascular risk. In this sense, there are other examples of associations between *PVRL2* variants and lipid parameters in other types of cohorts. For instance, rs6859 in *PVRL2* has been recently associated with LDLc levels in subjects with mild cognitive impairment. This association was also related to the risk of developing AD [51].

In a Korean general population cohort, it was found that a haplotype comprising five genetic variants, which included rs403155 in *PVRL2*, had a strong association with hyper-LDL cholesterolemia risk [52]. In a general population cohort of Chinese origin, 12 SNPs in the cluster of *BCL3*-*PVRL2*-*TOMM40* were studied, and haplotype analysis showed that they are associated with lipid parameters [53]. Most of the previously reported associations between *PVRL2* variants and lipid parameters are based on cholesterol, and we are describing associations with parameters highly linked to TG metabolism. Our results are probably influenced by the fact that the pleiotropy analysis was conducted with data from a GWAS focusing on TG, but further studies are needed to confirm this relationship.

PVRL2 is a single-pass type I membrane glycoprotein that is one of the plasma membrane components of adherent junctions [54], and it plays an important role in the pathogenesis of atherosclerosis by regulating the transendothelial migration of leukocytes [55,56]. Additionally, it is a cholesterol-responsive gene that acts at endothelial sites of vascular inflammation due to its ability to modulate the transendothelial migration of leukocytes, and animal models deficient in *PVRL2* have less atherosclerosis [57]. How PVRL2 is related to the lipid profile characteristic of atherogenic dyslipidemia remains elusive; thus, further studies are needed.

This study has some limitations that need to be acknowledged. The first limitation is the sample size, which may be limited for genetic association studies. However, the fact that previous studies have described similar associations in other populations gives value to our results, even though our results were obtained in small cohorts. *PVRL2* is proximal to *APOE*, which is a key gene in lipid metabolism, and it may have a significant influence on surrounding genes. Thus, to avoid confounding the described associations by proximity to *APOE*, we adjusted our analysis by *APOE* genotype.

The present study focused on *PVRL2*; however, other genes and SNPs have also been identified that may need further study to confirm their relevance in the predisposition of the cardiometabolic profile.

In conclusion, we have identified three variants in *PVRL2* that predispose individuals to AD and that influence the lipid profile that confers cardiovascular risk in T2DM subjects. Thus, we describe a potential new modulating factor of atherogenic dyslipidemia that should be considered when assessing cardiovascular risk in the future.

## Figures and Tables

**Table 1:** Baseline characteristics of cognitive impairment cohort.

	MCI, n = 146	AD, n = 184	All, n = 330	*p*
Age, years	75.00 ± 8	78.00 ± 7	76.00 ± 7	<0.001
Female sex, %	63.7	70.7	67.6	NS
ApoE4 carriers, %	43.8	50.3	47.4	0.023
MMSE test score, a.u.	23.54 (4.41)	17.20 (5.62)	20.20 (5.95)	<0.001
Type 2 diabetes, %	12.3	14.4	13.3	NS
Glucose, mmol/L	5.78 (2.00)	5.87 (1.96)	5.87 (1.99)	NS
RLP cholesterol, mg/dL	8.67 ± 4.86	9.05 ± 5.50	8.93 ± 5.18	NS
Total cholesterol, mmol/L	5.58 (1.02)	6.20 (5.23)	5.94 (3.91)	NS

Data are presented as the median ± interquartile range for continuous variables with a nonnormal distribution and number (percentage) for categorical variables. MMSE; Mini Mental State Examination. RLP; remnant-like particles.

**Table 2:** SNPs associated with RLPc levels in the cognitive impairment cohort.

SNP (Gene)		n	Median ± IQR	*p*
rs1789072 (in *DSC1*)	Homozygotes of common allele	315	9.44 ± 6.56	0.047
	Carriers of rare allele	164	8.41 ± 5.85	
rs3025786 (in *PSEN1*)	Homozygotes of common allele	402	9.44 ± 6.59	0.012
	Carriers of rare allele	77	7.90 ± 5.34	
rs73572039 (in *PVRL2*)	Homozygotes of common allele	369	9.57 ± 6.75	0.009
	Carriers of rare allele	101	8.28 ± 4.56	
rs3788428 (in *PLA2G3*)	Carriers of common allele	471	9.18 ± 6.18	0.015
	Homozygotes of rare allele	3	4.94 ± 2.57	
rs1049296 (in *TF*)	Carriers of common allele	451	9.31 ± 6.57	0.050
	Homozygotes of rare allele	26	7.77 ± 3.06	

IQR: interquartile range.

**Table 3:** Baseline characteristics of the high cardiovascular risk population.

	All, n = 281
Age, years	56.08 (11.26)
Female sex, %	134 (47.3)
Body mass index, kg/m^2^	31.45 (6.66)
Type 2 diabetes, %	189 (66.8)
Glucose, mmol/L	7.38 (2.84)
RLP cholesterol, mg/dL	10.27 ± 10.81
Total cholesterol, mmol/L	5.64 (1.39)
LDL cholesterol, mmol/L	3.47 (1.20)
HDL cholesterol, mmol/L	1.45 (0.33)
Triglycerides, mmol/L	1.64 ±2.06
Apolipoprotein B100, mg/dL	107.75 (27.49)
Apolipoprotein AI, mg/dL	135.64 (12.96)

Data are presented as the mean (SD) for continuous variables with a normal distribution, median ± interquartile range for continuous variables with a nonnormal distribution, and number (percentage) for categorical variables. RLP, remnant-like lipoproteins; LDL, low-density lipoprotein; HDL, high-density lipoprotein.

**Table 4:** Associations of *PVRL2* SNPs with lipid and lipoprotein profile in high cardiovascular risk population.

	rs73572039			rs12978931			rs11667640			
	Homozygotes of Common Allele (n = 201)	Carriers of Rare Allele (n = 78)	*p*	Carriers of Common Allele (n = 263)	Homozygotes of Rare Allele (n = 11)	*p*	Homozygotes of Common Allele (n = 221)	Hetero (n = 52)	Homozygotes of Rare Allele (n = 2)	*p*
Plasma lipids										
Triglycerides, mmol/L	1.74 ±2.42	1.44 ±1.48	0.032	1.40 ± 1.90	2.20 ± 3.91	0.001	1.50 ± 2.00	1.40 ± 1.70	4.05 ± NA	NS
Cholesterol, mmol/L	5.66 (1.97)	5.54 (1.41)	NS	5.84 (1.36)	6.24 (1.92)	NS	5.85 (1.32)	5.91 (1.67)	6.10 (1.41)	NS
LDL cholesterol, mmol/L	3.40 (1.19)	3.58 (1.22)	NS	3.81 (1.14)	3.34 (1.24)	NS	3.74 (1.08)	4.01 (1.40)	3.53 (1.00)	NS
HDL cholesterol, mmol/L	1.44 (0.30)	1.46 (0.38)	NS	1.15 (0.38)	1.06 (0.55)	NS	1.17 (0.41)	1.08 (0.25)	0.85 (0.42)	0.006
RLP cholesterol, mg/dL	10.73 ± 12.45	9.63 ± 8.75	0.049	9.24 ± 7.57	12.72 ± 11.68	0.045	9.70 ± 8.75	8.80 ± 5.66	43.80 ± NA	0.030
ApoB100, mg/dL	106.91 (27.58)	109.05 (27.37)	NS	126.58 (37.67)	126.67 (45.53)	NS	125.26 (36.37)	132.50 (44.25)	116.00 (19.80)	NS
ApoAI, mg/dL	135.48 (13.02)	136.66 (12.94)	NS	142.81 (27.49)	143.40 (32.80)	NS	143.91 (28.31)	139.21 (25.09)	127.00 (21.21)	NS
Lipoprotein subclass										
VLDL-P, mmol/L	61.43 ±113.98	46.33 ±62.38	0.007	50.04 ± 99.80	157 ± 155.25	0.003	56.31 ± 108.96	41.93 ± 85.55	174.19 ± NA	NS
Large VLDL, mmol/L	2.13 ±2.73	1.55 ±1.84	0.009	1.89 ± 2.61	3.59 ± 4.34	0.003	2.11 ± 2.65	1.50 ± 2.33	5.68 ± NA	NS
Medium VLDL, mmol/L	8.95 ±14.57	6.99 ±9.59	0.007	7.84 ± 11.79	22.52 ± 19.67	0.003	8.44 ± 12.86	6.98 ± 10.31	24.89 ± NA	NS
Small VLDL, mmol/L	51.22 ±96.99	36.73 ±51.40	0.007	40.57 ± 85.06	131.30 ± 134.25	0.003	46.81 ± 89.06	34.01 ± 72.02	143.62 ± NA	NS
LDL-P, mmol/L	987.17 (426.99)	982.93 (363.43)	NS	1001.43 (405.63)	860.30 (498.17)	NS	990.35 (393.02)	1031.43 (471.42)	561.58 (282.28)	NS
Large LDL, mmol/L	123.52 (52.82)	126.81 (48.87)	NS	126.93 (51.39)	93.68 (56.27)	0.053	124.56 (49.28)	131.27 (61.46)	72.47 (462.3)	NS
Medium LDL, mmol/L	341.74 ±198.70	338.14 ±186.78	NS	371.94 ± 198.15	312.73 ± 205.65	NS	368.60 ± 188.36	362.02 ± 266.83	190.04 ± NA	NS
Small LDL, mmol/L	492.57 ±325.25	478.12 ±227.09	NS	516.64 ± 295.29	485.88 ± 533.75	NS	520.59 ± 280.33	557.69 ± 363.86	299.07 ± NA	NS
HDL-P, µmol/L	25.41 ±8.33	24.68 ±6.57	NS	25.05 ± 9.02	26.16 ± 13.32	NS	25.62 ± 9.75	24.18 ± 5.54	31.74 ± NA	NS
Large HDL, µmol/L	0.15 ±0.08	0.14 ±0.07	NS	0.14 ± 0.09	0.13 ± 0.19	NS	0.14 ± 0.09	0.15 ± 0.07	0.16 ± NA	NS
Medium HDL, µmol/L	6.70 ±4.62	6.59 ±3.58	NS	6.94 ± 4.87	6.28 ± 5.51	NS	6.82 ± 5.02	7.03 ± 4.66	8.01 ± NA	NS
Small HDL, µmol/L	18.24 ±5.81	17.16 ±5.89	0.016	17.94 ± 5.37	20.64 ± 7.74	0.039	18.36 ± 5.95	16.76 ± 3.70	23.56 ± NA	NS
Lipoprotein size										NS
VLDL size, nm	42.50 ±0.90	42.52 ±0.67	NS	42.53 ± 0.89	42.23 ± 0.97	NS	42.51 ± 0.91	42.70 ± 0.82	42.65 ± NA	NS
LDL size, nm	21.03 ±0.26	21.10 ±0.22	NS	21.08 ± 0.26	20.82 ± 0.43	<0.001	21.06 ± 0.28	21.10 ± 0.23	20.91 ± NA	NS
HDL size, nm	8.17 ±0.13	8.21 ±0.11	0.027	8.19 ± 0.13	8.12 ± 0.17	0.041	8.18 ± 0.14	8.22 ± 0.13	8.13 ± NA	

Data are presented as the mean (SD) for continuous variables with a normal distribution and median ± interquartile range for continuous variables with a nonnormal distribution. P, total particles; RLP, remnant-like lipoproteins; VLDL, very low-density lipoprotein; LDL, low density lipoprotein; HDL, high density lipoprotein.

## Data Availability

The data presented in this study are available on request from the corresponding author.

## Supplementary Materials

The following supporting information can be downloaded at: https://www.mdpi.com/article/10.3390/ijms24087415/s1.
