# Supplementary material for: Metabolic Overlap between Alzheimer’s Disease and Metabolic Syndrome Identifies the PVRL2 Gene as a New Modulator of Diabetic Dyslipidemia
Source: Int J Mol Sci. 2023 Apr 18;24(8):7415. doi: 10.3390/ijms24087415 (PMC10139078; doi:10.3390/ijms24087415)
Supplement: Supplementary file 1 [file ijms-24-07415-s001.zip › ijms-2271757-SI.pdf]

SUPPLEMENTAL TABLE

Supplemental Table S1. List of genes and SNPs associated with AD.

Chr	Symbol	Gene Name	SNP Id	MAF_{ceu}
1	*CHRNB2*	Cholinergic receptor, nicotinic, beta polypeptide 2	rs4845378	0.10
1	*DHCR24*	24-dehydrocholesterol reductase	rs2274941	0.43
1	*MTHFR*	Methylenetetrahydrofolate Reductase	rs1801131	0.31
1	*NCSTN*	Nicastrin	rs12239747	0.05
2	*EIF2AK2*	Eukaryotic Translation Initiation Factor 2A	rs2254958	0.43
			rs4648174	0.41
			rs3770768	0.13
			rs1805164	0.31
3	*TF*	Transferrin	rs1049296	0.14
4	*IGFBP7*	Insulin Like Growth Factor Binding Protein	rs4075349	0.42
5	*HMGCR*	3-Hydroxy-3-Methylglutaryl-CoA Reductase	rs5909	0.10
			rs3761740	0.10
5	*HMGCS1*	3-Hydroxy-3-Methylglutaryl-CoA Synthase 1	rs12514393	0.47
6	*ESR1*	Estrogen Receptor 1	rs3844508	0.13
6	*TAP2*	Transporter 2, ATP Binding Cassette Subfamily B Member	rs241448	0.28
6	*TTBK1*	Tau Tubulin Kinase 1	rs1995300	0.32
			rs2651206	0.33

7	*DNAJB9*	DNAJ Heat Shock Protein Family (Hsp40) Member B9	rs2227272	0.20
8	*YWHAZ*	Tyrosine 3-Monooxygenase/Tryptophan 5-Monooxygenase Activation Protein Zeta	rs983583	0.41
9	*GOLM1*	Golgi Membrane Protein 1	rs10868366	0.09
9	*HSPA5*	Heat Shock Protein Family A (Hsp70) Member 5	rs17840761	0.46
10	*CALHM1*	Calcium homeostasis modulator 1	rs41287502	0.02
10	*SORCS1*	Sortilin Related VPS10 Domain Containing Receptor 1	rs601883	0.11
10	*TFAM*	Transcription Factor A, Mitochondrial	rs2306604	0.44
			rs11006130	0.17
			rs2279340	0.23
			rs10826177	0.22
11	*HYOU1*	Hypoxia Up-Regulated 1	rs13929	0.40
12	*DDIT3*	DNA damage inducible transcript 3	rs3847699	0.18
12	*IGF1*	Insulin Like Growth Factor 1	rs17727841	0.17
			rs1019731	0.13
			rs4764697	0.27
			rs2195240	0.23
14	*PSEN1*	Presenilin 1	rs3025786	0.06
14	*SEL1L*	SEL1L ERAD E3 Ligase Adaptor Subunit	rs12436488	0.21
16	*MAP1LC3B*	Microtubule Associated Protein 1 Light Chain 3 Beta	rs11117269	0.48
16	*TMC5*	Transmembrane Channel Like 5	rs2245086	0.16
17	*MAPT*	Microtubule Associated Protein Tau	rs1864325	0.24

18	*DSC1*	Desmocollin 1	rs1789072	0.19
19	*GAPDHS*	Glyceraldehyde-3-Phosphate Dehydrogenase, Spermatogenic	rs4806173	0.39
19	*PVRL2*	Poliovirus related receptor 2	rs73572039	0.13
			rs3745150	0.44
20	*PRNP*	Prion Protein	rs1799990	0.33
22	*HMOX1*	Heme Oxygenase 1	rs2071746	0.44
22	*PLA2G3*	Phospholipase A2 group 3	rs2232170	0.21
			rs9619169	0.48
			rs3788428	0.09
			rs2074739	0.43

MAF_{CEU}: minor allele frequency in populations of European ancestry.

Supplemental Table S2. List of genomic loci associated with both AD and TG.

locusnum	snpid	geneid	chrnum	chrpos	pval_AD	fdr_AD	conjfdr_AD_TriG	prune_AD_TriG	min_conjfdr
1	rs7528604	PDE4B	1	66407352	$2,13 \times 10^{-4}$	$1,66 \times 10^{-1}$	$4,93 \times 10^{-2}$	1	$4,93 \times 10^{-2}$
2	rs1994077	SYN2	3	12117220	$8,83 \times 10^{-4}$	$3,42 \times 10^{-1}$	$3,47 \times 10^{-2}$	1	$3,47 \times 10^{-2}$
3	rs7712360	ANKRD55	5	55780101	$3,71 \times 10^{-4}$	$2,26 \times 10^{-1}$	$3,65 \times 10^{-2}$	1	$3,65 \times 10^{-2}$
4	rs1019457	ZNF366	5	71763154	$9,35 \times 10^{-4}$	$3,51 \times 10^{-1}$	$3,64 \times 10^{-2}$	1	$3,64 \times 10^{-2}$
5	rs4574536	HSPA4	5	132384689	$5,06 \times 10^{-4}$	$2,63 \times 10^{-1}$	$4,66 \times 10^{-2}$	1	$4,66 \times 10^{-2}$
6	rs6995541	SOX7	8	10671260	$9,39 \times 10^{-4}$	$3,52 \times 10^{-1}$	$3,65 \times 10^{-2}$	1	$3,65 \times 10^{-2}$
7	rs10093055	SH2D4A	8	19046110	$6,67 \times 10^{-4}$	$3,00 \times 10^{-1}$	$2,73 \times 10^{-2}$	1	$2,73 \times 10^{-2}$
8	rs17405319	TRIB1	8	126449406	$6,22 \times 10^{-4}$	$2,90 \times 10^{-1}$	$2,56 \times 10^{-2}$	1	$2,56 \times 10^{-2}$
9	rs1015109	TRIB1	8	126590593	$2,79 \times 10^{-4}$	$1,94 \times 10^{-1}$	$1,24 \times 10^{-2}$	1	$1,24 \times 10^{-2}$
10	rs1883025	ABCA1	9	107664301	$3,92 \times 10^{-4}$	$2,32 \times 10^{-1}$	$1,69 \times 10^{-2}$	1	$1,69 \times 10^{-2}$
11	rs676309	AB231731	11	60001573	$3,34 \times 10^{-10}$	$2,41 \times 10^{-6}$	$3,43 \times 10^{-2}$	1	$3,43 \times 10^{-2}$
12	rs7976512	STAC3	12	57643686	$7,83 \times 10^{-4}$	$3,23 \times 10^{-1}$	$3,13 \times 10^{-2}$	1	$3,13 \times 10^{-2}$
13	rs4930724	LIMKAIN	12	124423817	$9,64 \times 10^{-4}$	$3,56 \times 10^{-1}$	$3,73 \times 10^{-2}$	1	$3,73 \times 10^{-2}$
14	rs12708454	LIPC	15	58692202	$2,74 \times 10^{-5}$	$4,28 \times 10^{-2}$	$1,38 \times 10^{-3}$	1	$1,38 \times 10^{-3}$
15	rs1017545	RAB8B	15	63553994	$5,15 \times 10^{-7}$	$1,74 \times 10^{-3}$	$2, \times 10^{-4}$	1	$2,68 \times 10^{-4}$

16	rs966062	UBN1	16	4907430	$2,39 \times 10^{-4}$	$1,77 \times 10^{-1}$	$4,89 \times 10^{-2}$	1	$4,89 \times 10^{-2}$
17	rs1549293	KAT8	16	31141993	$2,92 \times 10^{-6}$	$7,43 \times 10^{-3}$	$1,58 \times 10^{-4}$	1	$1,58 \times 10^{-4}$
18	rs8075803	FLJ40194	17	47346529	$7,21 \times 10^{-6}$	$1,53 \times 10^{-2}$	$3,82 \times 10^{-4}$	1	$3,82 \times 10^{-4}$
19	rs4803750	BCL3	19	45247627	$5,14 \times 10^{-28}$	$8,19 \times 10^{-7}$	$1,55 \times 10^{-7}$	1	$1,55 \times 10^{-7}$
20	rs12978931	PVRL2	19	45363700	$3,79 \times 10^{-32}$	$8,19 \times 10^{-7}$	$1,14 \times 10^{-3}$	1	$1,14 \times 10^{-3}$
20	rs11667640	PVRL2	19	45379791	$1,44 \times 10^{-16}$	$8,19 \times 10^{-7}$	$1,15 \times 10^{-8}$	1	$1,15 \times 10^{-8}$
20	rs157580	TOMM40	19	45395266	$5,41 \times 10^{-217}$	$8,19 \times 10^{-7}$	$1,15 \times 10^{-8}$	1	$1,15 \times 10^{-8}$
21	rs3760627	CLPTM1	19	45457180	$1,72 \times 10^{-9}$	$1,07 \times 10^{-5}$	$1,01 \times 10^{-7}$	1	$1,01 \times 10^{-7}$
22	rs676388	FUT2	19	49211969	$9,77 \times 10^{-4}$	$3,58 \times 10^{-1}$	$3,77 \times 10^{-2}$	1	$3,77 \times 10^{-2}$
